# Hospitalization for tuberculosis in children and adolescents: clinical, epidemiological characteristics and post-treatment sequelae at a referral center in Brazil

**DOI:** 10.1016/j.bjid.2026.105890

**Published:** 2026-07-09

**Authors:** Ana Lucia Nunes Diniz, Nelbe Nesi Santana Avzaradel, Saint Clair dos Santos Gomes Júnior

**Affiliations:** Instituto Nacional de Saúde da Mulher, da Criança e do Adolescente Fernandes Figueira, Fundação Oswaldo Cruz (Fiocruz), Rio de Janeiro, RJ, Brazil

**Keywords:** Tuberculosis, Child, Adolescent, Hospitalization, Sequelae, Post-tuberculosis

## Abstract

**Background:**

Tuberculosis in children may require hospitalization and can be associated with the development of sequelae even after completion of antituberculosis treatment, with potential long-term impacts on the health of children and adolescents.

**Methods:**

This observational, longitudinal study was based on the review of medical records of children and adolescents hospitalized for tuberculosis at a referral center in Brazil. Clinical and epidemiological characteristics, care pathways, and the occurrence of post-treatment sequelae were described. Bivariate and multivariable analyses were performed to identify factors associated with the development of sequelae.

**Results:**

A total of 87 patients were included, predominantly of mixed race, of both sexes, with a median age of 6-years. After hospital discharge, 92.0% remained under follow-up at the referral center. Among these patients, 88.8% achieved a cure and 11.2% discontinued treatment. Among patients who were cured, 46.5% developed sequelae after treatment completion. The presence of sequelae was associated with age ≥ 10-years, pulmonary tuberculosis, and markers of greater disease severity during hospitalization, including the need for invasive mechanical ventilation and longer length of hospital stay.

**Conclusions:**

Despite the high cure rate, a substantial proportion of children and adolescents developed post-tuberculosis sequelae. These findings highlight the need for a comprehensive approach, including post-treatment and multidisciplinary follow-up, centered on children and adolescents, to reduce morbidity and long-term functional impairment across the life course.

## Introduction

Tuberculosis remains one of the leading causes of morbidity and mortality from infectious diseases worldwide, including in Brazil. Although a declining trend has been observed in recent years, the country continues to report a high incidence of tuberculosis.^1^ In 2023, an estimated 10.8 million new cases were reported globally, approximately 12% of which occurred among children aged 0–14 years.^2^ Tuberculosis in children and adolescents reflects not only the intensity of community transmission but also underlying social vulnerabilities and barriers to timely access to healthcare services.^2^^,^^3^

In settings with a high incidence of tuberculosis, hospitalization is a frequent event.^4^ and, in the pediatric population, is considered a marker of disease severity. Hospital admission is often associated with severe clinical forms, such as miliary, meningoencephalitic, or disseminated tuberculosis. In addition, the presence of comorbidities, including HIV infection, malnutrition, and genetic or immunological conditions, may further exacerbate disease severity. Delayed diagnosis and limited access to primary healthcare services significantly contribute to the need for hospitalization, which frequently occurs in the context of nonspecific clinical presentations, making differentiation from other respiratory diseases challenging.^5^, ^6^, ^7^, ^8^, ^9^, ^10^

National and international studies have shown that hospitalization of children and adolescents with tuberculosis is associated with an increased risk of mortality and post‑treatment sequelae, including functional impairment and reduced pulmonary function, even among those who achieve treatment success. In cases of miliary and meningoencephalitic tuberculosis, mortality rates remain high, and up to 49% of survivors may develop neurological sequelae, such as epilepsy and motor, cognitive, or hearing déficits.^6^^,^^11^, ^12^, ^13^, ^14^, ^15^, ^16^ Despite the epidemiological relevance of tuberculosis in pediatric populations, substantial knowledge gaps persist regarding factors associated with hospitalization disease severity, and clinical outcomes in the Brazilian contexto.^17^ The scarcity of longitudinal studies limits the development and implementation of effective strategies for prevention, early diagnosis, and comprehensive long-term management of tuberculosis in children and adolescents.

Therefore, considering hospitalization as an indicator of disease severity and a potential predictor of sequelae, and recognizing the importance of understanding the clinical and epidemiological characteristics of tuberculosis in the pediatric population to inform public health policies and improve care protocols, this study aimed to describe the clinical and epidemiological characteristics of children and adolescents hospitalized for tuberculosis at a referral center in Brazil and to identify factors associated with the development of post-tuberculosis sequelae.

## Materials and methods

### Study design, setting, and population

We conducted an observational, longitudinal study involving children and adolescents hospitalized with a diagnosis of tuberculosis at a high-complexity referral center of the Brazilian Unified Health System (Sistema Único de Saúde ‒ SUS) in the state of Rio de Janeiro, Brazil, from January 2009 to December 2023.

### Inclusion and exclusion criteria

We included medical records of patients aged ≤ 18-years with confirmed pulmonary, extrapulmonary, or mixed tuberculosis, who were admitted for treatment or diagnostic investigation. Medical records thar were unavailable at the time of data collection were excluded, as were readmissions of the same patient, only the first eligible admission was considered.

### Data collection and variables

Data were obtained through standardized retrospective review of medical records using a structured data collection form and stored in REDCap (Research Electronic Data Capture). Information was organized into three time points: hospital admission, inpatient period, and post-discharge follow-up.

At hospital admission, we collected sociodemographic characteristics, Bacillus Calmette-Guérin (BCG) vaccination status, history of previous contact with tuberculosis, prior diagnostic investigation, reason for hospitalization, presence of comorbidities, clinical signs and symptoms, and time from symptom onset to diagnosis. During hospitalization, data included diagnostic methods, clinical form of tuberculosis, HIV testing results, Tuberculin Skin Test (TST) results, radiographic findings, need for Intensive Care Unit (ICU) admission, ventilatory support, length of hospital stay, antituberculosis treatment regimen, and in-hospital outcomes.

In the post-discharge period, we assessed referrals to other specialties, need for hospital readmission, duration of anti-tuberculosis treatment, pharmacological treatment completion (cure, loss to follow-up, or death), and the presence of sequelae after treatment completion.

### Definitions

BCG vaccination status was confirmed by the presence of a characteristic vaccination scar on the right arm and/or documentation in the vaccination card record. Tuberculosis diagnosis was based on laboratory confirmation, clinical-epidemiological criteria, or the diagnostic scoring system recommended by the Brazilian Ministry of Health. A positive Tuberculin Skin Test (TST) was defined as an induration of ≥ 5 mm. Laboratory confirmation included sputum smear microscopy, culture for Mycobacterium tuberculosis, rapid molecular testing (GeneXpert® MTB/RIF), and histopathology.

### Outcome

The primary outcome was the presence of post-tuberculosis sequelae identified at or after completion of the anti-tuberculosis treatment. Post-tuberculosis sequelae were defined as any abnormalities detected at treatment completion or up to the last recorded follow-up visit among patients classified as cured. Sequelae included pulmonary abnormalities identified by chest imaging, ventilatory impairment demonstrated by pulmonary function testing, neurofunctional deficits (motor or sensory), and other sequelae documented in the medical record. The presence of at least one of these findings was considered a positive outcome.

### Statistical analysis

Statistical analyses were performed using JASP software (version 0.19.3, JASP Team, 2024). Continuous variables were summarized as medians and minimum ‒ maximum ranges, and categorical variables were presented as absolute and relative frequencies. Analysis of post-tuberculosis sequelae was restricted to patients classified as cured at anti-tuberculosis treatment completion, as outcome assessment occurred at the end of the treatment.

Associations between categorical variables and the presence of sequelae were evaluated using Pearson’s Chi-Square test or Fisher’s exact test when expected cell counts were < 5. Variables with p-values < 0.20 in bivariate analysis were included in a multivariable logistic regression model. Results are presented as adjusted Odds Ratios (OR) with 95% Confidence Intervals (95% CI). A two-sided p-value < 0.05 was considered statistically significant.

### Ethics

The study protocol was submitted to and approved by the Research Ethics Committee of the referral center (CAAE: 78,726,424.8.0000.5269), under approval number 6.773.914.

## Results

**Fig. 1:**
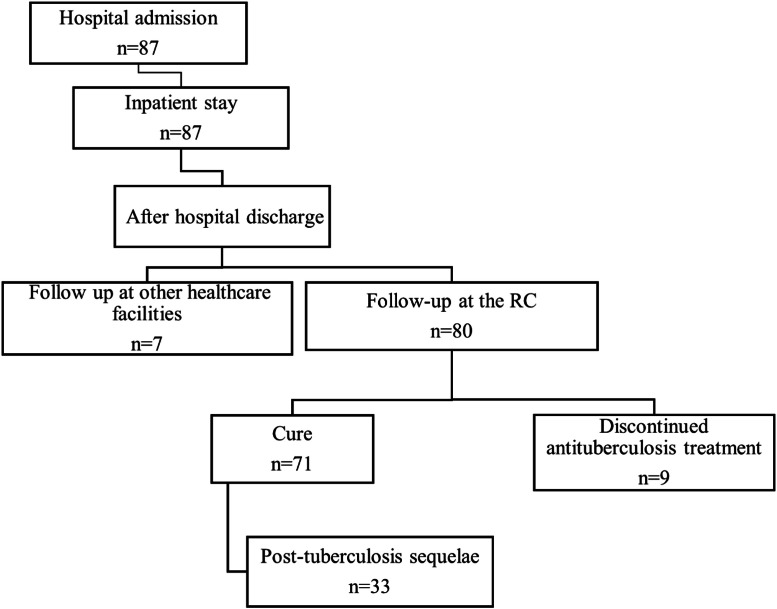
Flowchart of the patient pathway from hospital admission to post-discharge follow-up and outcomes. RC, Referral Center.

During the study period, 87 medical records were included, with no missing data related to hospital admission or inpatient stay (Fig. 1). After hospital discharge, seven patients (8.0%) continued outpatient follow-up at other healthcare facilities, whereas 80 patients (92.0%) remained under follow-up at the referral center. Among those followed at the referral center, 71 patients (88.8%) achieved a cure and 9 (11.2%) discontinued antituberculosis treatment.

Among patients who achieved a cure (*n* = 71), 28 (39.4%) continued to receive clinical follow-up at the referral center after anti-tuberculosis treatment completion. Post-tuberculosis sequelae were documented in 33 patients (46.5%) up to the last recorded visit following completion of the anti-tuberculosis regimen.

At hospital admission, most patients were of mixed race (80.5%), with a balanced distribution between sexes and a median age of 6-years (range, 0–17 years). In most cases, the reason for hospitalizations was not directly attributed to tuberculosis (83.9%), mainly nonspecific lung diseases (46.0%) and hospitalizations for diagnostic investigation of tuberculosis (17.2%). Although 53 patients (60.9%) reported previous contact with a bacilliferous tuberculosis case, only 21.8% had undergone prior contact tracing.

Most patients were previously healthy (74.7%). Among those with comorbidities, HIV infection was the most frequent accounting for 14 of 22 cases. The most commonly reported symptoms at admission were fever (77.0%), cough (54.0%), and weight loss (26.4%). The most frequent clinical signs included reduced or absent vesicular breath sounds (58.6%), adventitious lung sounds (36.8%), and tachypnea (33.3%). The median time from symptom onset to hospitalization was 20-days (range, 0–566-days). The clinical and demographic characteristics of children and adolescents with tuberculosis at hospital admission are presented in Table 1.

**Table 1:** Characteristics of children and adolescents with tuberculosis at hospital admission.

Characteristics	n (%) or median (min – max)
Gender (male)	44 (50.6)
Age (years)	6 (0 ‒ 17)^a^
Race/ethnicity	
Mixed race	70 (80.5)
White	8 (9.2)
Black	7 (8.0)
Other	2 (2.3)
Age group ≥ 10-years	35 (40.2)
BCG vaccination (yes)	81 (93.1)
Previous contact with TB (yes)	53 (60.9)
Previous TB investigation (yes)	19 (21.8)
Reason for hospitalization not related to TB^b^	73 (83.9)
Lung disease	40 (46.0)
Diagnostic investigation for TB	15 (17.2)
Other causes	18 (20.7)
Comorbidities (yes)	22 (25.3)
Symptoms^b^	
Fever	67 (77.0)
Cough	47 (54.0)
Weight loss	23 (26.4)
Abdominal pain	10 (11.5)
Chest pain	10 (11.5)
Headache	8 (9.2)
No symptoms	2 (2.3)
Other	39 (44.8)
Clinical signs^b^	
Reduced or absent vesicular breath sounds	51 (58.6)
Adventitious lung sounds	32 (36.8)
Tachypnea	29 (33.3)
Lymphadenopathy	12 (13.8)
Meningeal signs	4 (4.6)
No abnormalities	3 (3.4)
Other	19 (21.8)
Time from symptom onset to hospitalization (days)	20 (0‒566)^a^

BCG, Bacilo Calmette-Guérin; TB, Tuberculosis.

a Median (minimum – maximum).

b Variables represent multiple responses; frequencies are not mutually exclusive, and percentages do not total 100%.

During hospitalization, tuberculosis was diagnosed by laboratory confirmation in 44 patients (50.6%) and by clinical-epidemiological assessment using the Brazilian Ministry of Health diagnostic scoring system in 42 patients (48.3%), other diagnostic approaches were used in one patient (1.1%). Among laboratory methods, culture was the most frequently performed test (65.9%), followed by sputum smear microscopy for acid-fast bacilli (52.3%), rapid molecular testing (Xpert MTB/RIF) (36.4%), and histopathology (29.5%). More than one diagnostic test was performed per patient when applicable. Two cases of Multidrug-Resistant Tuberculosis (MDR-TB) were identified (2 cases; 2.3%).

The Tuberculin Skin Test (TST) was performed in 62 patients (71.3%), with a reactive result observed in 33 (53.2%). Information on HIV testing was available in 82 medical records (94.3%), and tuberculosis-HIV coinfection was identified in 15 patients (18.3%), including cases in which HIV infection was diagnosed during hospitalization.

The pulmonary clinical form was the most frequent clinical presentation (40 patients; 46.0%), followed by exclusive extrapulmonary disease (25 patients; 28.7%) and mixed pulmonar and extrapulmonary forms (22 patients; 25.3%). Among patients with extrapulmonary involvement (exclusive extrapulmonary and mixed forms; *n* = 47), pleural (15 patients; 31.9%) and lymph node disease (14 patients; 29.8%) predominated, followed by meningoencephalitic (8 patients; 17.0%), disseminated (5 patients; 10.6%), and mediastinal forms (4 patients; 8.5%). More than one extrapulmonary site could be identified in the same patient.

Chest radiographs were available for 79 patients, and abnormalities were reported in most cases. The most frequent radiographic findings were pulmonary consolidations (43 patients; 54.4%), pleural effusion (20 patients; 25.3%), atelectasis (12 patients; 15.2%), and cavitary lesions (9 patients; 11.4%). No radiographic abnormalities were identified in 12 patients (15.2%).

Regarding inpatient management, 22 patients (25.3%) required admission to the Intensive Care Unit (ICU), and nine of these patients (40.9%) required invasive mechanical ventilation. The median total length of hospital stay was 16-days (range, 1–144 days), and the median ICU stay was 4-days (range, 1–31 days). Post-discharge complications were recorded in 6.4% of cases, and all patients were discharged from the hospital. Inpatient clinical characteristics of children and adolescents with tuberculosis are summarized in Table 2.

**Table 2:** Characteristics of children and adolescents with tuberculosis during hospitalization.

Characteristics	n (%) or median (min – max)
Diagnostic method	
Laboratory confirmation^b^	44 (50.6)
Culture	29 (65.9)
BAAR	23 (52.3)
RMT-TB	16 (36.4)
Histopathology	13 (29.5)
Clinical-epidemiological assessment/Scoring system	42 (48.3)
Other	1 (1.1)
TST performed	62 (71.3)
TST (Reactive)	33 (53.2)
HIV test recorded	82 (94.3)
HIV positive (TB-HIV coinfection)	15 (18.3)
Clinical form	
Pulmonary TB	40 (46.0)
Exclusive extrapulmonary TB	25 (28.7)
Mixed TB	22 (25.3)
Extrapulmonary TB site (*n* = 47)^b^	
Pleural	15 (31.9)
Lymph node	14 (29.8)
Meningoencephalitic	8 (17.0)
Disseminated	5 (10.6)
Mediastinal	4 (8.5)
Other	5 (10.6)
Chest radiographs available^b^	79 (90.8)
Homogeneous or heterogeneous consolidations	43 (54.4)
Pleural effusion	20 (25.3)
Atelectasis	12 (15.2)
Cavitations	9 (11.4)
Adenomegaly	6 (7.6)
Other	18(22.8)
No abnormalities	12 (15.2)
ICU admission	22 (25.3)
Invasive mechanical ventilation (among ICU patients, *n* = 22)	9 (40.9)
Length of ICU stay (days)	4 (1‒31)^a^
Total length of hospital stay (days)	16 (1‒144)^a^

TST Reactive percentages were calculated among patients who underwent TST (*n* = 62).

Percentages for extrapulmonary TB sites were calculated among patients with extrapulmonary involvement (*n* = 47).

Radiographic findings were calculated among patients with available chest radiographs (*n* = 79).

TST, Tuberculin Skin Test; HIV, Human Immunodeficiency Virus; TB, Tuberculosis; ICU, Intensive Care Unit; BAAR, Sputum smear microscopy for acid-fast bacill; RMT-TB, Rapid Molecular Testing.

a Median (minimum – maximum).

b Variables represent multiple responses; frequencies are not mutually exclusive, and percentages do not total 100%.

During outpatient follow-up, among the 80 patients who remained under care at the referral center, 32 (40.0%) required referral to other healthcare specialties. During the course of treatment, 13 patients (16.3%) required hospital readmission. The observed cure rate was 88.8% (71-patients), while nine patients (11.2%) discontinued treatment, and no deaths were recorded.

Oh the 71 patients cured patients, 24 (33.8%) showed no abnormalities on chest radiographs performed after completion of anti-tuberculosis treatment. The most frequently observed persistente radiographic abnormalities were homogeneous or heterogeneous pulmonary consolidations (11.3%), followed by atelectasis (5.6%). Overall, 33 cured patients (46.5%) developed post-tuberculosis sequelae, predominantly pulmonary, followed by neurofunctional impairments and other conditions documented in their medical records. In addition, 28 patients continued outpatient follow-up in at least one medical specialty at the referral center.

The median duration of anti-tuberculosis treatment was 6-months (range, 6–14 months), regardless of the clinical form of tuberculosis. Outpatient follow-up characteristics of children and adolescents after hospitalization are presented in Table 3.

**Table 3:** Outpatient follow-up after hospital discharge of children and adolescents with tuberculosis.

Variable	n (%) or median (min–max)
Continuity of care^b^	
Directly observed therapy	7 (8.0)
Family Health Clinic	6 (6.9)
Other	8 (9.2)
Outpatient clinic at the RC	80 (92.0)
Referral to other healthcare specialties (*n* = 80)	32 (40.0)
Hospital readmission (*n* = 80)	13 (16.3)
TB treatment completion (*n* = 80)	
Cure	71 (88.8)
Treatment abandonment	9 (11.2)
Death	0
Post-tuberculosis sequelae (*n* = 71)^b^	33 (46.5)
Pulmonary	26 (36.6)
Neurofunctional	6 (8.5)
Other	4 (5.6)
Duration of antituberculosis treatment (months)	6 (6‒14)^a^

TB, Tuberculosis; RC, Referral Center.

a Median (minimum – maximum).

b Variables represent multiple responses; frequencies are not mutually exclusive, and percentages do not total 100%.

In the bivariate analysis, the presence of post-tuberculosis sequelae was more frequent among patients aged ≥ 10 years (54.5%vs. 28.9%; *p* = 0.03), those presenting with reduced or absent vesicular breath sounds at hospital admission (78.8%vs. 50.0%; *p* = 0.01), patients with positive sputum smear microscopy (33.3%vs. 10.5%; *p* = 0.02), and those who required invasive mechanical ventilation during hospitalization (21.2%vs. 2.6%; *p* = 0.01). A significant association was also observed between sequelae and longer hospital length of stay (median, 20 vs. 11 days; *p* = 0.04).

In contrast, the frequency of sequelae was lower among male patients (36.4%vs. 65.8%; *p* = 0.01), and no cases of sequelae were identified among patients whose diagnosis was established by histopathological examination (*p* = 0.001). Variables with a p-value < 0.20 in the bivariate analysis are presented in Table 4.

**Table 4:** Bivariate analysis of factors associated with post-tuberculosis sequelae among children and adolescents.

Factor	No sequelae, n (%) (*n* = 38)	With sequelae, n (%) (*n* = 33)	p
Age ≥ 10-years	11 (28.9)	18 (54.5)	0.03
Gender male	25 (65.8)	12 (36.4)	0.01
Reason for hospitalization related to TB (TB vs. NTB)	2 (5.3)	7 (21.2)	0.07
Cough (yes)	18 (47.4)	22 (66.7)	0.10
Reduced or absent vesicular breath sounds (yes)	19 (50.0)	26 (78.8)	0.01
Lymphadenopathy (yes)	7 (18.4)	1 (3.0)	0.06
TST (Reactive)	18 (47.4)	10 (30.3)	0.14
Positive sputum smear microscopy (yes)	4 (10.5)	11 (33.3)	0.02
Positive culture (yes)	9 (23.7)	13 (39.4)	0.15
Histopathological diagnosis (yes)	10 (26.3)	0 (0.0)	0.001
Clinical form			0.13
Pulmonary TB	12 (31.6)	16 (48.5)	
Exclusive extrapulmonary TB	15 (39.5)	6 (18.2)	
Mixed TB	11 (28.9)	11 (33.3)	
Extrapulmonary TB site			
Lymph node	10 (26.3)	1(3.0)	0.14
Disseminated	1 (2.6)	4 (12.1)	0.18
Invasive mechanical ventilation (yes)	1 (2.6)	7 (21.2)	0.01
Total length of hospital stay (days)^a^	11 (1–65)	20 (3–144)	0.04

Categorical variables were compared using Pearson’s Chi-Square test or Fisher’s exact test. as appropriate; continuous variables were compared using the Mann-Whitney *U test*.

TB, Tuberculosis; NTB, Not Tuberculosis; TST, Tuberculin Skin Test.

a Data are presented as median (minimum – maximum).

In the multivariable logistic regression analysis, age > 10 years showed a trend toward higher odds of developing post-tuberculosis sequelae (OR = 2.68; 95% CI 0.92–7.81; *p* = 0.070). In contrast, male sex was independently associated with lower odds of the outcome (OR = 0.22; 95% CI 0.07–0.70; *p* = 0.010).

Regarding the clinical form of tuberculosis (reference category: exclusive extrapulmonary tuberculosis), pulmonary tuberculosis was associated with increases odds of developing sequelae (OR = 5.04; 95% CI 1.25–20.33; *p* = 0.023), whereas the mixed pulmonar and extrapulmonary form was not significantly associated with the outcome (OR = 2.35; 95% CI 0.60–9.24; *p* = 0.222) (Table 5).

**Table 5:** Multivariable logistic regression analysis of factors associated with post-tuberculosis sequelae among hospitalized children and adolescents.

Factor	Adjusted OR (95% CI)	p
Age ≥ 10-years	2.68 (0.92–7.81)	0.070
Gender male	0.22 (0.07–0.70)	0.010
Clinical form (reference: exclusive extrapulmonary TB)		
Pulmonary TB	5.04 (1.25–20.33)	0.023
Mixed TB	2.35 (0.60–9.24)	0.222

OR, Odds Ratio; 95% CI, Confidence Interval. Reference categories: < 10-years and female sex. The model was adjusted for age group, sex, and clinical form. Outcome: presence of sequelae (Yes = 1).

## Discussion

In this study, we described the care pathways of children and adolescents hospitalized for tuberculosis at a referral center and demonstrated that, even after completion of anti-tuberculosis treatment, patients classified as cured may develop post-tuberculosis sequelae requiring ongoing follow-up by multiple specialties in high-complexity healthcare settings. Although cure rates were high, a substantial proportion of patients experienced post-tuberculosis sequelae, particularly among older children, those with pulmonary form disease, and those presenting markers of greater clinical severity during hospitalization. These findings underscore the need for specialized follow-up beyond therapeutic discharge.

The cure rate observed in this study exceeded national and international tuberculosis control targets for the pediatric population,^18^^,^^19^ likely reflecting the high level of care provided in a specialized referral center with structured access to diagnosis, treatment, and follow-up. Nevertheless, the proportion of treatment discontinuation (11.2%) remains relevant and highlights the need for strategies at strengthening in care, particularly during the transition from hospital to output settings.

Despite favorable cure outcomes, nearly half of the patients developed post-tuberculosis sequelae, most commonly pulmonary. This finding reinforces that microbiological cure does not necessarily equate to full functional recovery, especially in cases with delayed diagnosis or more severe disease at presentation.^6^ Persistent radiological and functional abnormalities may adversely affect quality of life and physical development, supporting the importance of longitudinal post-treatment follow-up in pediatric tuberculosis care.^11^

The higher frequency of sequelae observed among patients aged ≥10-years may reflect disease patterns more similar to those seen in adults, characterized by higher bacillary burden, more extensive pulmonary involvement, and increased risk of permanent structural damage.^14^ Adolescents also more likely to experience diagnostic delays, whether due to lower initial clinical suspicion or barriers to accessing healthcare services, potentially resulting in greater tissue damage before treatment initiation.^7^^,^^15^

The association between pulmonary clinical form and increased odds of sequelae in multivariable analysis highlights the impact of direct parenchymal involvement on long-term outcomes. Extensive consolidations, atelectasis, and cavitary lesions may evolve into fibrosis, bronchiectasis, and persistent ventilatory impairment despite adequate treatment.^6^ Previous studies have demonstrated that significant and sustained loss of lung function can occur following pulmonary tuberculosis in childhood, reinforcing the need for systematic respiratory monitoring in the post-treatment period.^20^ Consistent with these findings, other investigations have reported substantial ventilatory deficits and impaired quality of life among children who complete treatment for pulmonary tuberculosis, supporting prolonged follow-up and comprehensive post-tuberculosis care.^13^

On the other hand, exclusive extrapulmonary disease was associated with a lower likelihood of sequelae in our analysis, although this finding should be interpreted cautiously. Non-pulmonary forms, particularly neurological tuberculosis, may still lead to significant long-term impairments that were not fully captured in this study. The small number of patients with neurological involvement limited more detailed subgroup analyses. Nevertheless, tuberculous meningitis in childhood remains associated with high mortality and substantial neurodevelopmental morbidity, even in specialized settings, underscoring the need for optimized treatment strategies and structured long-term follow-up.^21^ Pediatric tuberculous meningitis shows a high rate of sequelae and mortality, even in high-resource settings, and early diagnosis, dose optimization, and multidisciplinary management are essential to improve outcomes. Follow-up strategies should include periodic neurodevelopmental assessment, multidisciplinary care involving neurology, pulmonology, and rehabilitation services, as well as psychosocial support and evaluation of functional and quality-of-life outcomes.

Markers of greater clinical severity during hospitalization, such as the need for invasive mechanical ventilation and longer length of hospital stay, were associated with post-tuberculosis sequelae in bivariate analysis. These findings align with international evidence indicating that severe forms of pediatric tuberculosis requiring intensive support carry a higher risk of long-term structural and functional sequelae.^20^^,^^22^ Collectively, these results suggest that disease severity at presentation plays a central role in the development of sequelae and further emphasize the importance of early diagnosis and timely initiation of treatment to prevent long-term impairment.

The lower odds of sequelae observed among male patients, even after multivariable adjustment, should be interpreted with caution. Although sex-related differences in tuberculosis presentation have been described.^23^ the underlying mechanisms remain incompletely understood. In addition to biological factors, differences in healthcare access, health-seeking behavior, and treatment adherence may also contribute to observed disparities.^24^ Given the observational design and sample size, this finding should be considered exploratory.

Similarly, the absence of sequelae among patients diagnosed exclusively through histopathological examination may reflect chance findings related to the small number of cases rather than a true protective effect, reinforcing the limited statistical power of certain subgroup analyses.

From a healthcare delivery perspective, the high proportion of referrals to other specialties and the frequency of readmissions during treatment illustrate the complexity of managing hospitalized pediatric tuberculosis cases. These findings support the need for integrated, multidisciplinary care models and improved coordination between levels of care during post-treatment follow-up. Structured programs incorporating respiratory, neurological, and psychosocial evaluation may facilitate early detection and management of post-tuberculosis sequelae, in line with World Health Organization recommendations.^25^

Tuberculosis does not end with microbiological cure. In pediatric populations, the disease can result in significant long-term sequelae, including impaired lung development, neurofunctional deficits, and substantial psychosocial impact.^16^^,^^20^^,^^26^ However, data specifically addressing post-tuberculosis outcomes in children and adolescents remain limited, highlighting an important gap in the literature.

In adult populations, post-treatment assessment is recommended and typically includes pulmonary function testing, imaging studies, and multidisciplinary follow-up programs extending for up to two years, often incorporating pulmonary rehabilitation.^27^^,^^28^ In this context, adapting these strategies to pediatric populations, structured longitudinal monitoring and the use of standardized tools for functional and quality-of-life assessment may facilitate early identification of sequelae and improve long-term outcomes.^6^ Although evidence in children remains limited, post-treatment assessment should generally follow similar principles to those applied in adults. Pulmonary function testing should be considered, particularly in children aged 4- to 6-years with severe pulmonary involvement, while exercise capacity can be assessed in children aged ≥4-years using the six-minute walk test.^29^ Furthermore, quality-of-life assessment tools may be applied, with appropriate cultural adaptations, to better capture the broader impact of the disease in younger age groups. Moreover, integrating these approaches into tuberculosis control programs may promote more patient-centered care and strengthen the overall impact of public health policies.^25^

This study has limitations inherent to its retrospective and observational design, which precludes causal inference and introduces potential residual confounding. Data collection relied on the quality and completeness of medical records and the potential underestimation of mild or undocumented sequelae. The relatively small sample size may have limited the statistical power and reduced the precision of some estimates in the multivariable analysis, particularly in subgroups such as invasive mechanical ventilation, histopathological diagnosis, and multidrug-resistant tuberculosis. In addition, selection bias may have occurred, as only hospitalized patients were included, likely representing more severe cases of tuberculosis. Furthermore, as data were collected retrospectively from medical records, some important assessments could not be systematically performed, including respiratory functional evaluations, neurocognitive assessments, and quality-of-life measures potentially leading to underestimation of post-tuberculosis morbidity. Variability in follow-up duration may also have influenced the detection of late sequelae, and residual confounding cannot be excluded, as variables such as socioeconomic status, nutritional status, access to care, and treatment adherence were not systematically available. Despite these limitations, this study provides clinically relevant insights into post-tuberculosis outcomes in a high-risk pediatric population, supported by longitudinal follow-up in a specialized setting.

In summary, our findings demonstrate that even in a setting with high cure rates, a substantial burden of post-tuberculosis sequelae persists among children and adolescents. Older age (≥ 10-years), pulmonary form disease, and markers of greater clinical severity during hospitalization were associated with increased risk of sequelae. Pediatric tuberculosis care should therefore extend beyond microbiological cure, incorporating structure post-treatment and multidisciplinary follow-up focused on early detection and management of sequelae to reduce functional impairment and morbidity across the life course. Systematic evaluation of respiratory and neurological outcomes should be integrated into childhood tuberculosis control programs.

## Ethical approval

The study protocol was submitted to and approved by the Research Ethics Committee of the Fernandes Figueira Institute/Fiocruz (Plataforma Brasil), under CAAE number 78,726,424.8.0000.5269 and approval opinion number 6773,914, in accordance with Brazilian National Health Council Resolution n° 466/2012. Written informed consent was obtained from parents or legal guardians, and assent was obtained from participants when applicable. All data were analyzed confidentially and anonymized.

### Authors’ contributions

Ana Lucia Nunes Diniz: Conceptualization; Study design; data collection; data analysis and interpretation; drafting of the manuscript.

Nelbe Nesi Santana Avzaradel: Data analysis and interpretation; critical revision of the manuscript.

Saint Clair dos Santos Gomes Júnior: Data analysis and interpretation; manuscript revision.

All authors approved the final version of the manuscript and take responsibility for the accuracy and integrity of the work.

### Use of generative artificial intelligence

Generative artificial intelligence tools (M365 Copilot) were used exclusively for language revision of the manuscript. The use of AI did not affect the data, analyses, or interpretation of results. All authors reviewed and approved the final version of the manuscript.

### Originality and exclusivity

This manuscript is original, has not been previously published, and is not under consideration by another journal.

### Study origin

This article is derived from the ongoing PhD thesis entitled “Evaluation of tuberculosis care pathways: a series of hospitalized patients in a tertiary pediatric referral center between 2009 and 2023″, conducted by Ana Lucia Nunes Diniz within the Graduate Program in Applied Research on Child and Women’s Health at IFF/Fiocruz.

### Funding

This study received no specific funding from public, commercial, or not-for-profit funding agencies.

### Data availability statement

The data supporting the findings of this study are available from the corresponding author upon reasonable request.

## Conflicts of interest

The authors declare no conflicts of interest.
